# Supplementary material for: Heteroatom doping enables hydrogen spillover via H+/e− diffusion pathways on a non-reducible metal oxide
Source: Nat Commun. 2024 Jul 31;15:6403. doi: 10.1038/s41467-024-50217-z (PMC11291974; doi:10.1038/s41467-024-50217-z)
Supplement: Supplementary file 1 — Supplementary Information [file 41467_2024_50217_MOESM1_ESM.pdf]

Supporting Informations

Heteroatom Doping Enables Hydrogen Spillover via H^+/e^- Diffusion Pathways on a Non-reducible Metal Oxide

Kazuki Shun[1], Kohsuke Mori[1, 2*], Takumi Kidawara[1], Satoshi Ichikawa[3], and Hiromi Yamashita[1, 2]

[1] Division of Materials and Manufacturing Science, Graduate School of Engineering, Osaka University, 2-1 Yamada-oka, Suita, Osaka 565-0871, Japan. Tel & FAX: +81-6-6879-7460, +81-6-6879-7457

E-mail: mori@mat.eng.osaka-u.ac.jp

[2] Innovative Catalysis Science Division, Institute for Open and Transdisciplinary Research Initiatives (ICS-OTRI), Osaka University, Suita, Osaka 565-0871, Japan.

[3] Research Center for Ultra-High Voltage Electron Microscopy, Osaka University, Ibaraki 567-0047, Japan.

Table of Contents

Supplementary Note and Figures 2

References 26

Supplementary Note and Figures

Supplementary Note 1. Procedure used to determine the proportion of cation vacancies in octahedral sites in Al-MgO.

The proportion of the cation vacancies in octahedral sites (V_{Cat}) in the Al-MgO was determined using the following procedure. In this process, it was assumed that the Al-MgO was made of periclase (PR) and inversion spinel (IS) phases with fractions of *x* and *y*, and that these phases contained octahedral and tetrahedral Al (Al_{Oh} and Al_{Td}), respectively. The substitution of Mg ions in periclase MgO by Al_{Oh} will result in the formation of cation vacancies to maintain charge balance.[1] Considering that Mg and Al cations are divalent and trivalent, respectively, the substitution of Mg by Al_{Oh} can be summarized as $3\mathrm{Mg} \rightarrow 2\mathrm{Al_{Oh}} + \mathrm{V_{Oh}}$. If the Al_{Oh}/Mg molar ratio in the PR phase is defined as *z*, the stoichiometry of this phase will be:

$$Mg : Al_{Oh} : V_{Cat} : O = \frac{8}{2+3z} : \frac{8z}{2+3z} : \frac{4z}{2+3z} : 4. \quad (1)$$

In contrast, because the IS phase has a spinel structure,[2] the stoichiometry of this phase can be represented as:

$$Mg : Al_{Td} : V_{Cat} : O = 2 : 1 : 2 : 4. \quad (2)$$

The inductively coupled plasma atomic emission spectroscopy (ICP-AES) measurement (Fig. 1c) showed that the actual elemental ratios in the Al-MgO were:

$$(Mg + Al_{Oh} + Al_{Td}) : O = 48.2 : 51.8. \quad (3)$$

In addition, ^{27}Al solid-state nuclear magnetic resonance data indicated that the ratio between Al_{Oh} and Al_{Td} in the sample was:

$$Al_{Oh} : Al_{Td} = 80.8 : 19.2. \quad (4)$$

On the basis of equations (1) through (5), *x*, *y* and *z* were calculated to be 0.910, 0.090 and 0.120.

The proportion of V_{Cat} in the Al-MgO was subsequently calculated according to the formula:

$$\left(\frac{4z}{2+3z} \times x + 2 \times y\right)/4, \quad (5)$$

which gave a value of 0.092. The probability that octahedral cation sites adjacent to V_{Cat} are also empty can be calculated as

$$1-(1-0.092)^6=0.439. \quad (6)$$

Therefore, V_{Cat} is three-dimensionally extended with a probability of 43.9% within Al-MgO.

It should also be noted that no aggregation of the IS phase was believed to have occurred in the Al-MgO because this phase has been shown to grow epitaxially on periclase.[2] In addition, the XRD pattern of Al-MgO did not contain any diffractions other than those related to periclase MgO (**Fig. 1a**).

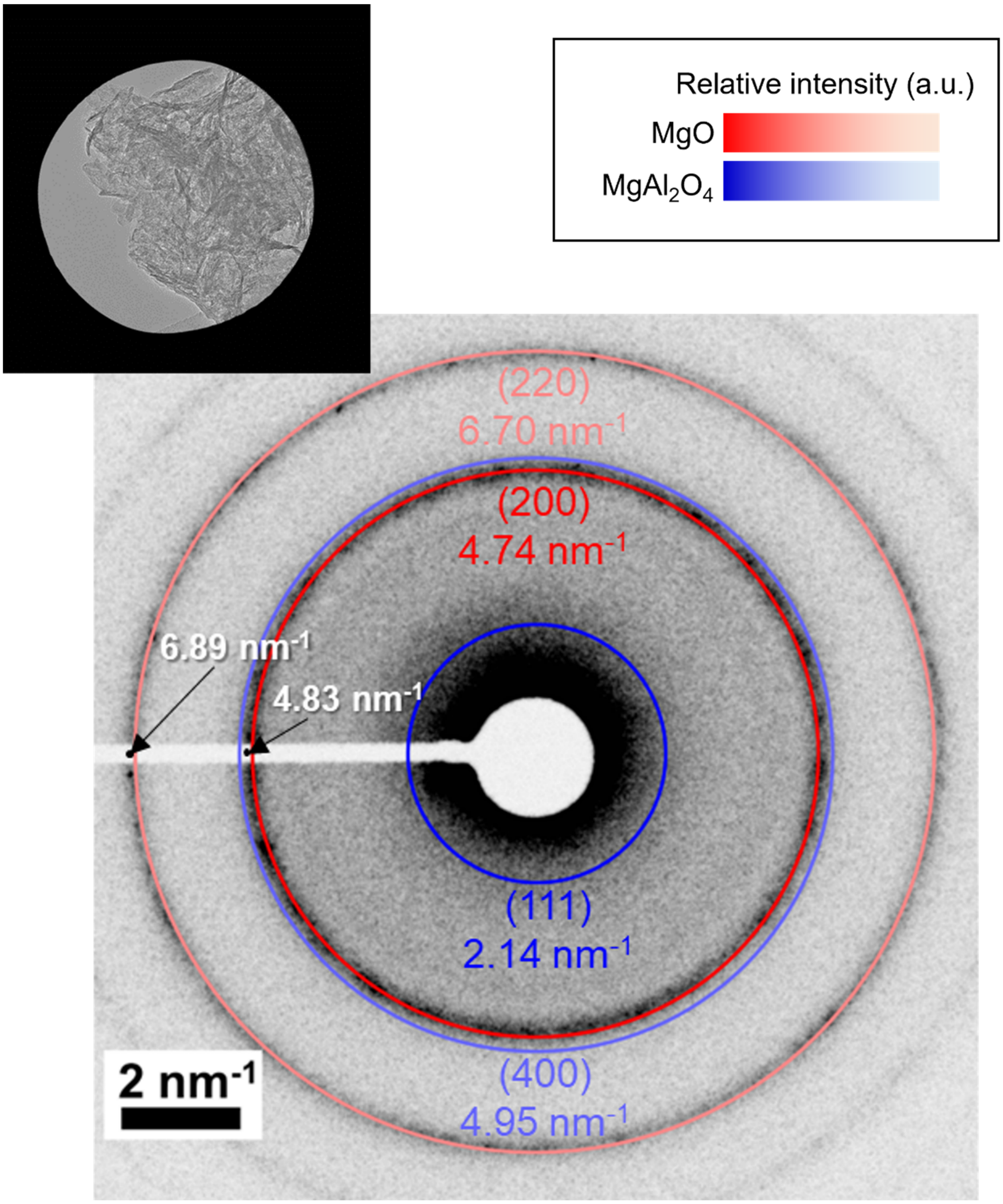

Supplementary Fig. 1. The local structure of Al-MgO.

Debye rings acquired from Al-MgO specimen within area shown in upper high resolution transmission electron microscopy image. The red and blue lines resulted from pristine MgO and $MgAl_2O_4$, respectively, based on simulations using the ReciPro software package.[3] In each case, the color intensity reflects the magnitude of the scattering factor for each facet.

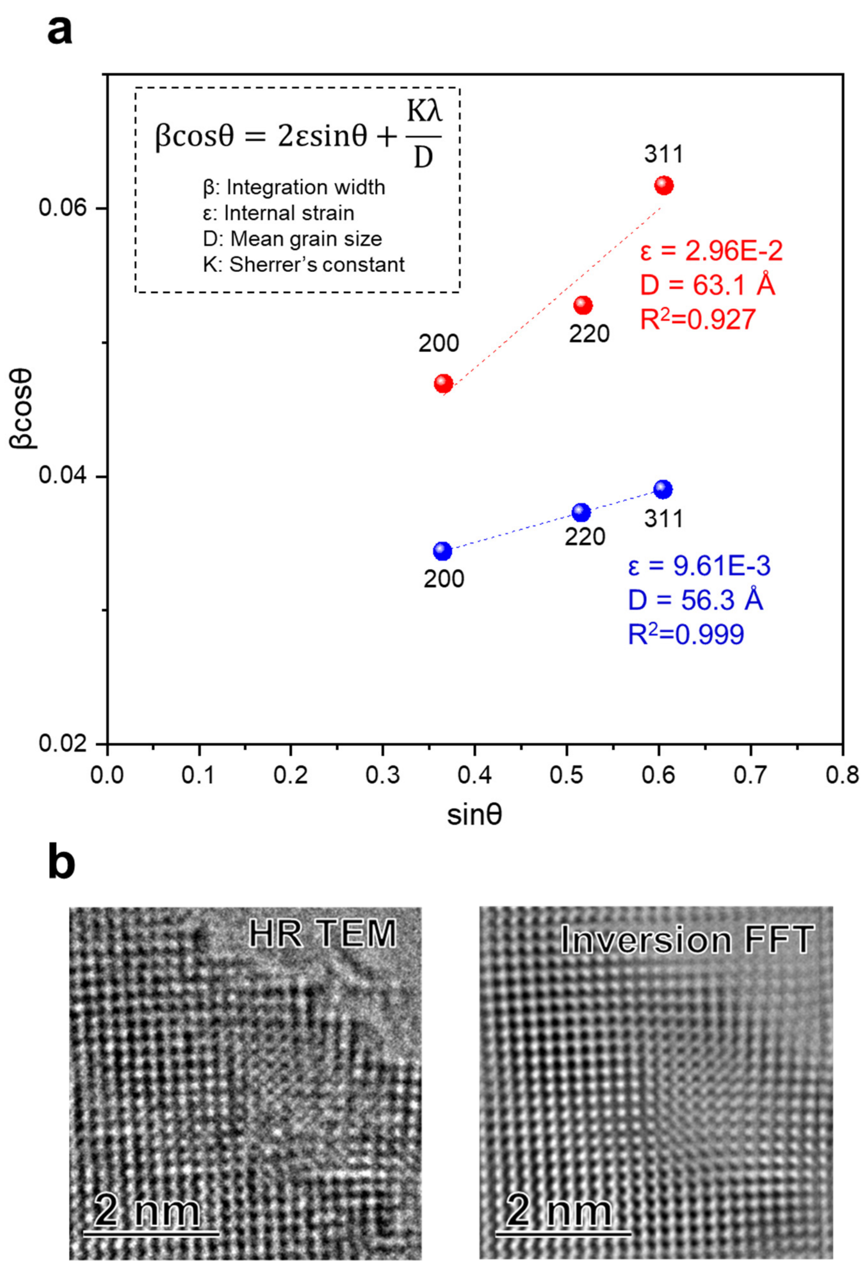

Supplementary Fig. 2. The internal strain generated within the Al-MgO.

a, Williamson-hall plots MgO (blue) and Al-MgO (red) obtained based on each XRD pattern.[4] The amount of internal strains (ε) and mean grain sizes (D) calculated from the slope and intercept of linear regressions were shown together, respectively. **b,** A HR TEM image and its inversion FFT image of Al-MgO obtained by using systematic diffraction spots of 001 and 010. The bending of lattice originated from internal strain was observed.

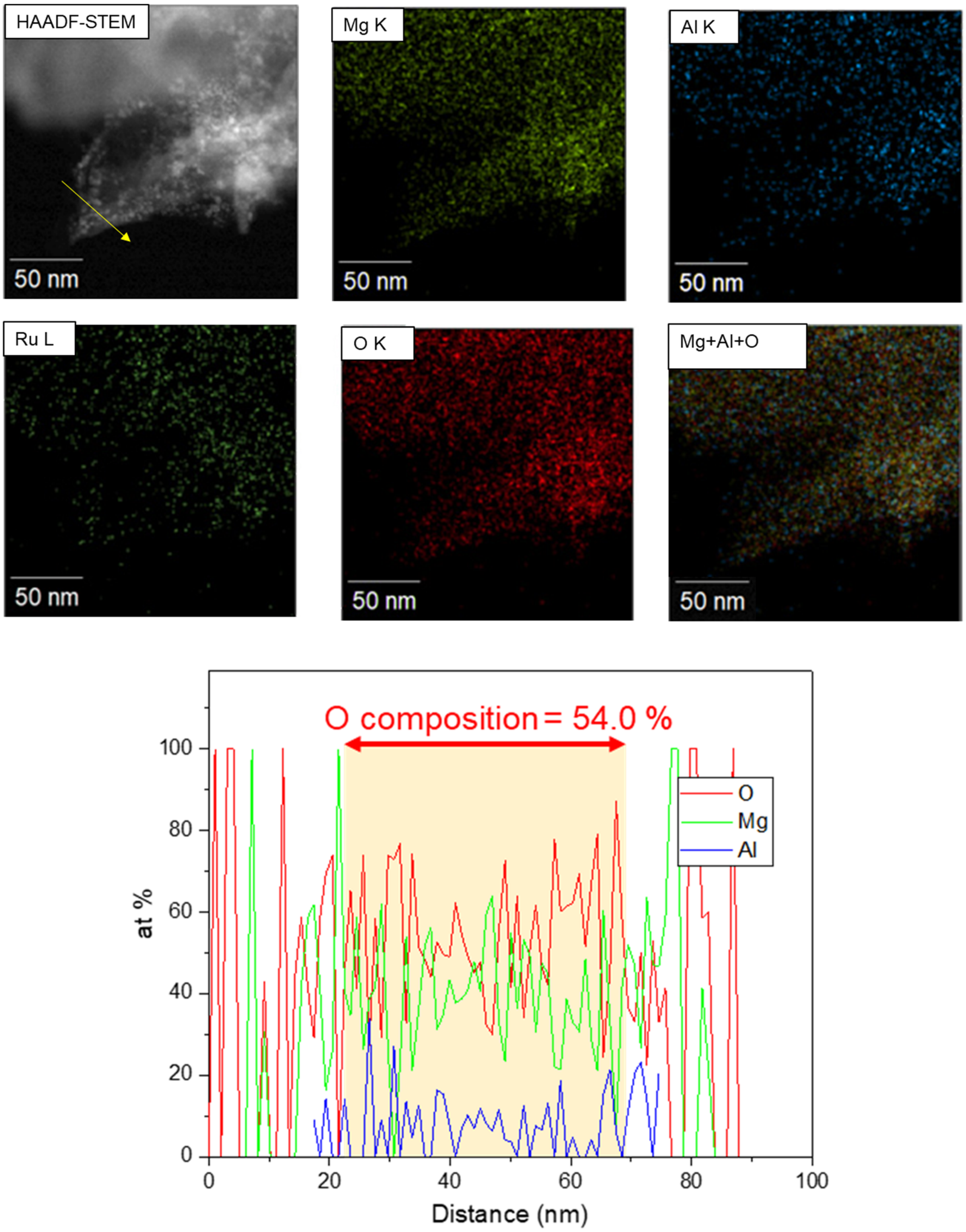

Supplementary Fig. 3. The elemental distribution within Al-MgO.

Top: a HAADF-STEM image and EDX maps obtained for the same field of view of Ru/Al-MgO. Bottom: the atomic composition of Al-MgO based on the EDX line analysis carried out along the path indicated by the yellow arrow shown in the HAADF-STEM image.

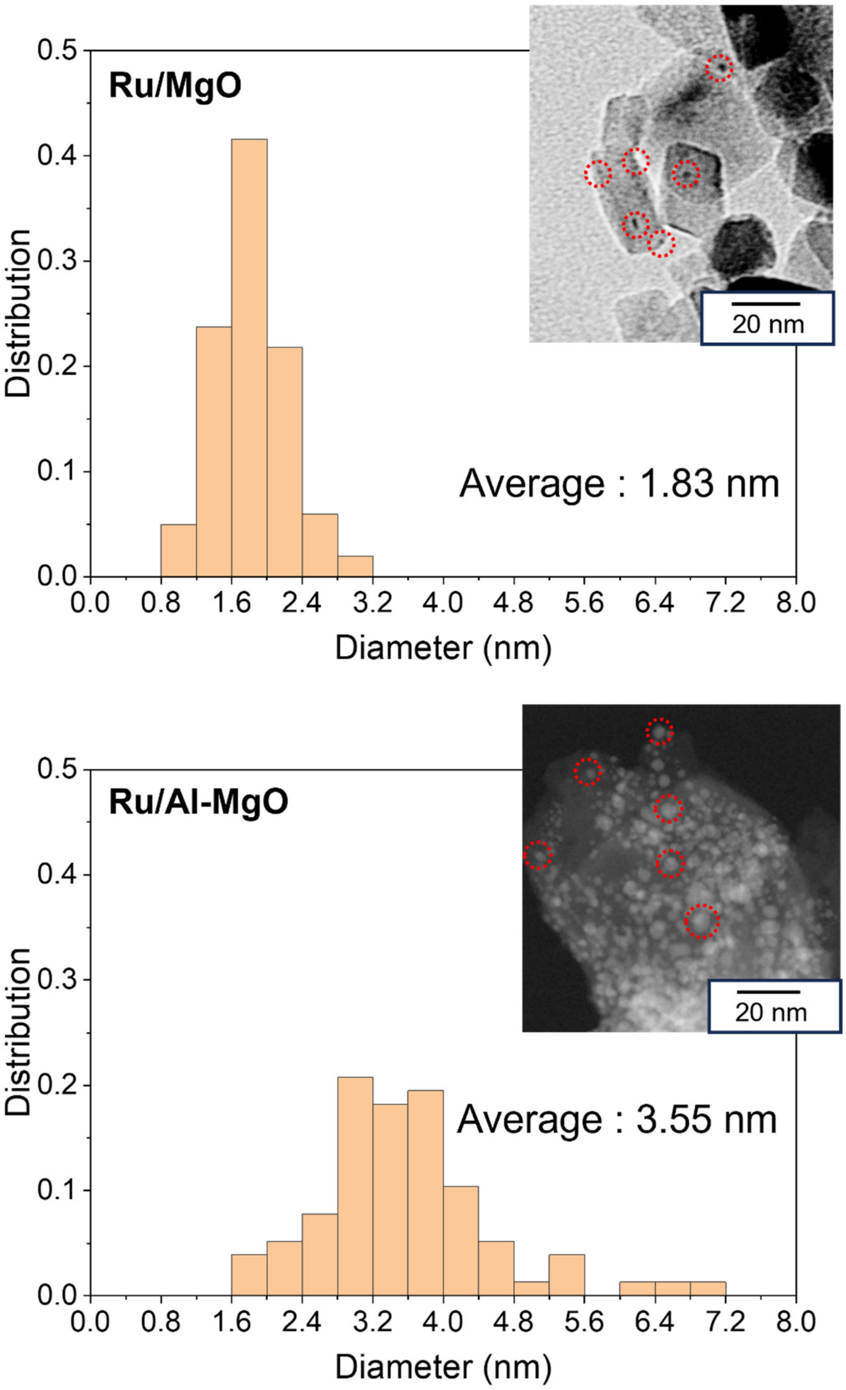

Supplementary Fig. 4. The evaluation of Ru nanoparticle size.

Size distributions of Ru nanoparticles deposited on Ru/MgO and Ru/Al-MgO as determined from transmission electron microscopy (TEM) and high-angle annual dark field scanning transmission electron microscopy (HAADF-STEM) images shown as inserts. The corresponding Ru nanoparticles are indicated in these images by red circles.

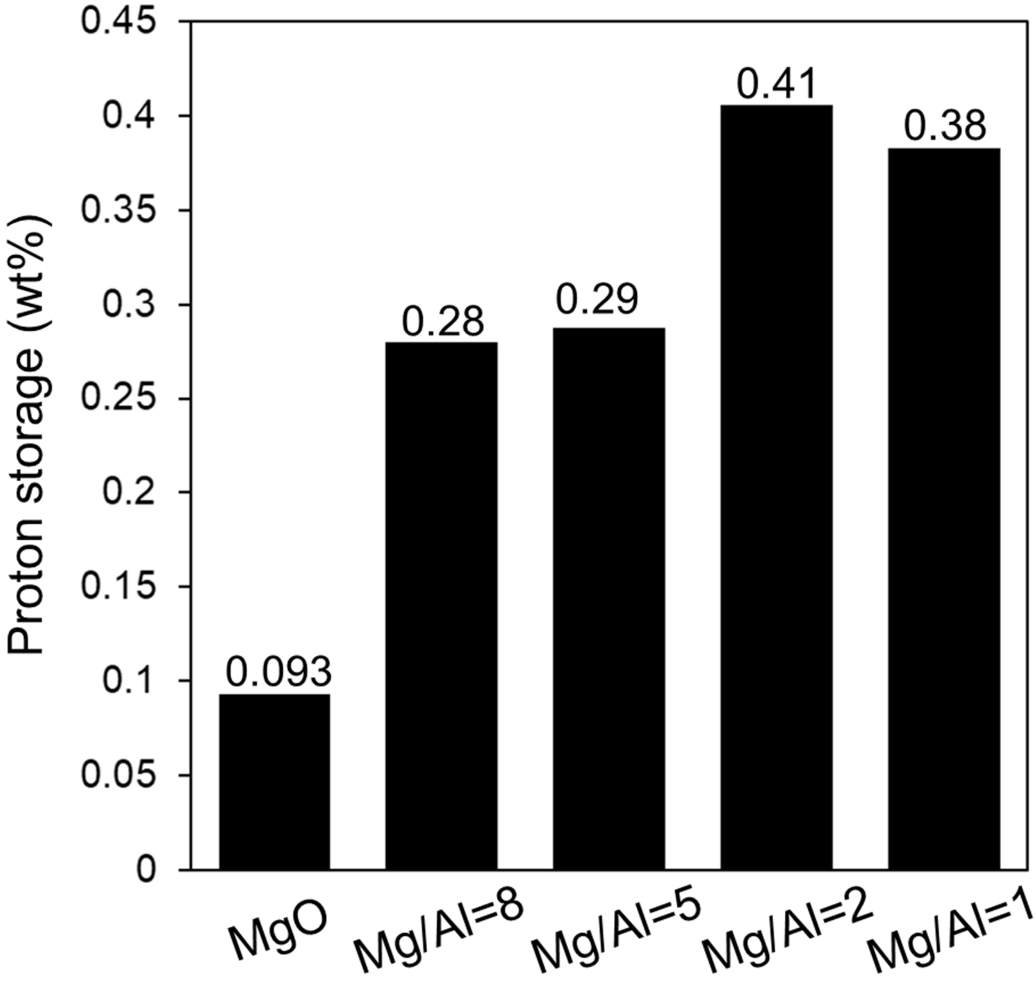

Supplementary Fig. 5. The effect of Mg/Al compositions on proton storage capacity.
The proton storage capacities of the Al doped MgO with different Mg/Al compositions evaluated by TG analyses while switching between H_2 and D_2 atmospheres at 400 °C.

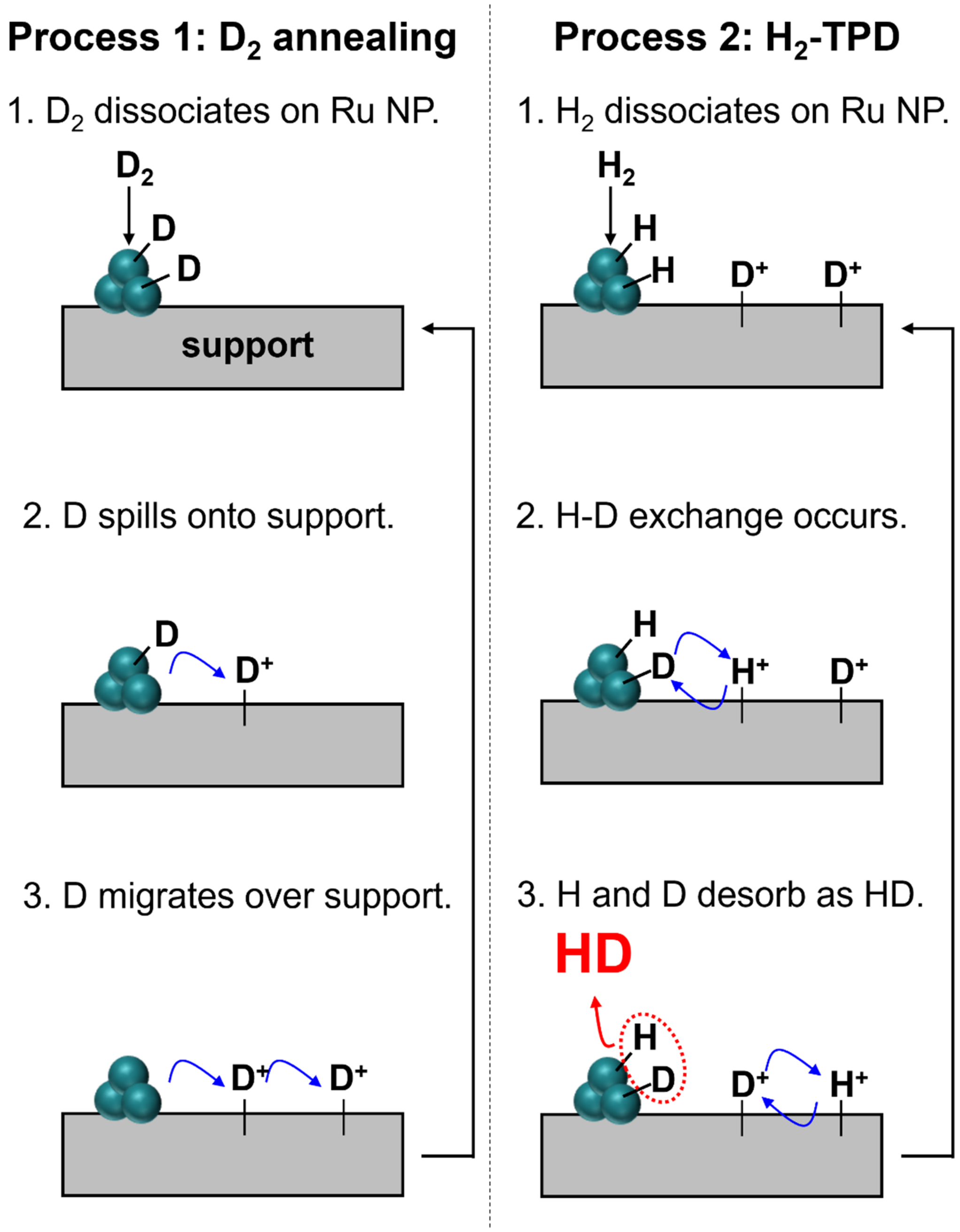

Supplementary Fig. 6. How HD molecules evolved during H_2-TPD followed by D_2 annealing. Mechanism for evolution of HD molecules via proton diffusion during H_2–temperature programmed desorption following annealing under D_2. The blue arrows indicate proton diffusion phenomenon.

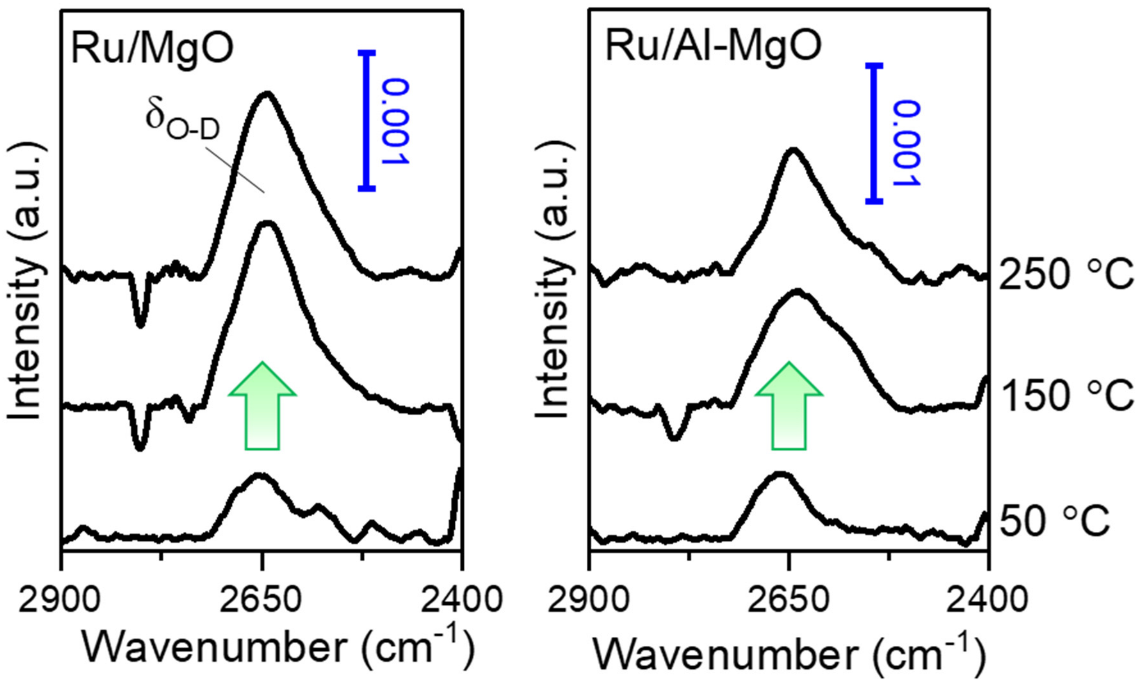

Supplementary Fig. 7. The observation of H^+ diffusion on the surface region.

In situ DRIFT spectra in a range of 2400 – 2900 cm^{-1} from Ru-loaded MgO and Al-MgO at various temperatures 10 min after switching to a D_2 flow. The vibrational stretching O–D bonds derived from D^+ diffusion on the surface (peaks in a range of 2600 – 2800 cm^{-1}) appeared from 50 to 150°C.[5]

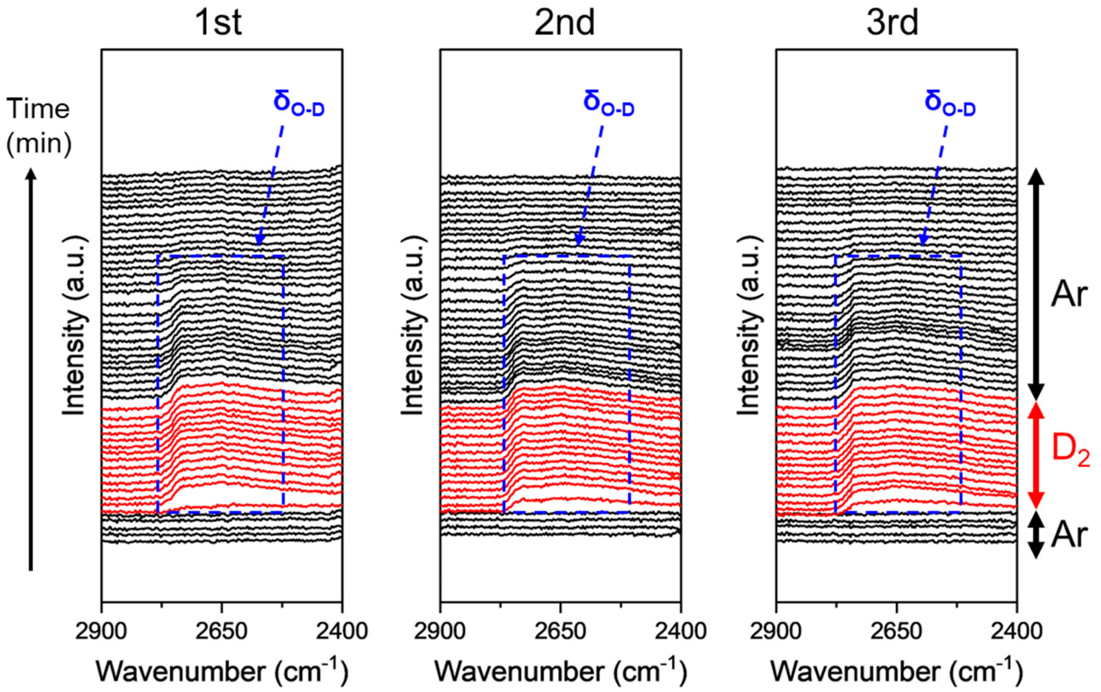

Supplementary Fig. 8. The reversible increase of the total concentration of surface O–D groups.

The transition of *in situ* DRIFT spectra from Ru/Al-MgO under atmospheres switching between Ar (black) and D_2 (red) at 600 °C. The DRIFT spectra were obtained every minute. The formation and disappear of the surface O–D groups was reproducibly confirmed.

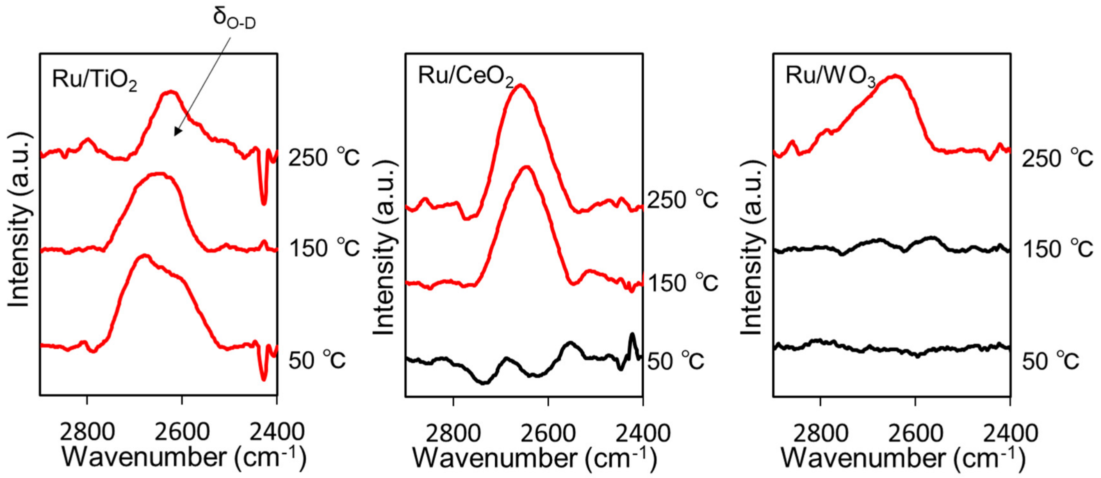

Supplementary Fig. 9. The observation of H^+ diffusion on the surface of reducible metal oxides.

In situ DRIFT spectra in a range of 2400 – 2900 cm^{-1} from Ru-loaded TiO_2, CeO_2, and WO_3 at various temperatures 10 min after switching to a D_2 flow.

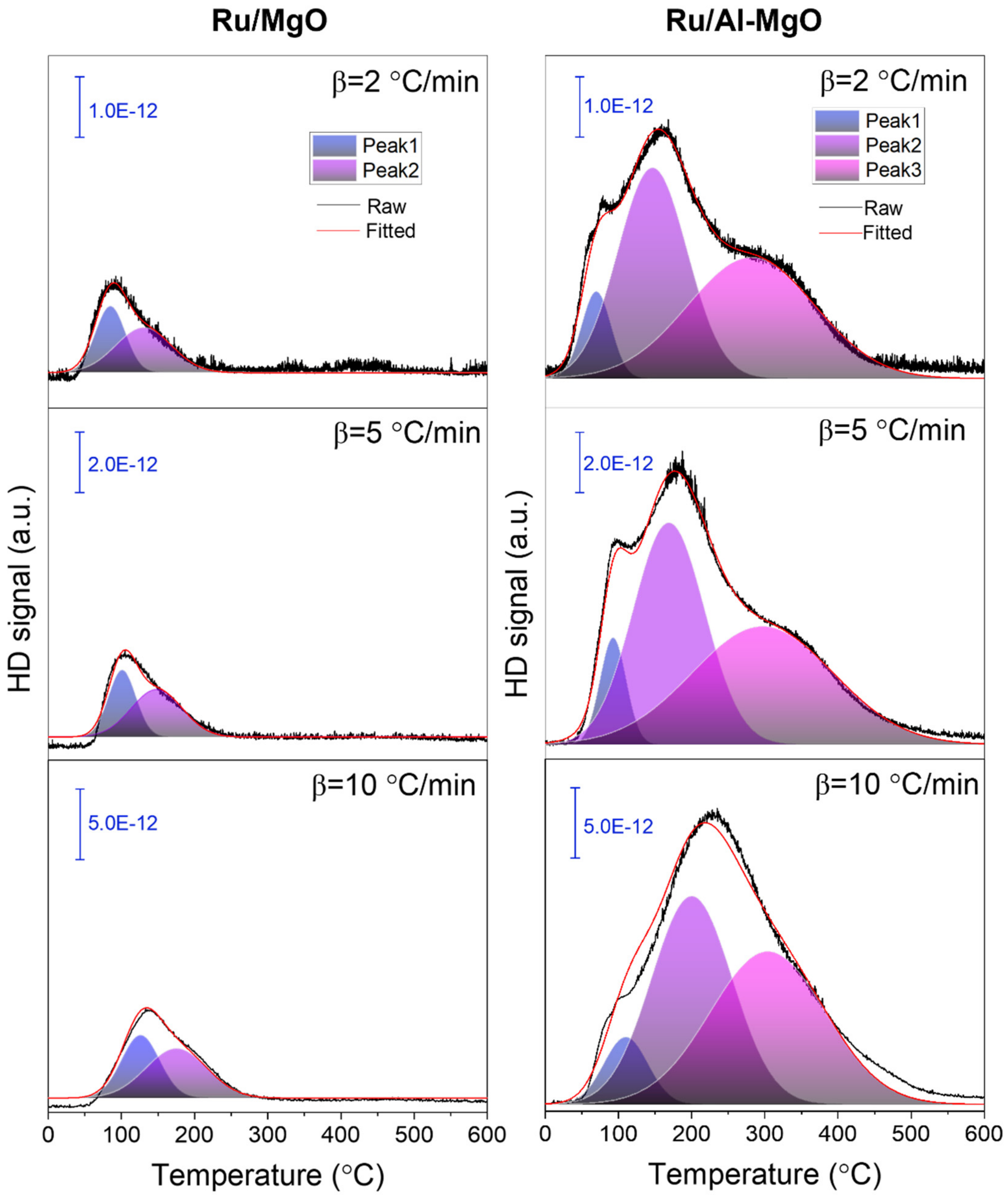

Supplementary Fig. 10. The effect of heating rates on the HD production.

HD production during H_2-temperature programmed reduction following D_2 annealing at various heating rates as determined using mass spectrometry for Ru/MgO (left) and Ru/Al-MgO (right) specimens. Each plot was deconvoluted to provide multiple Gaussian peaks.

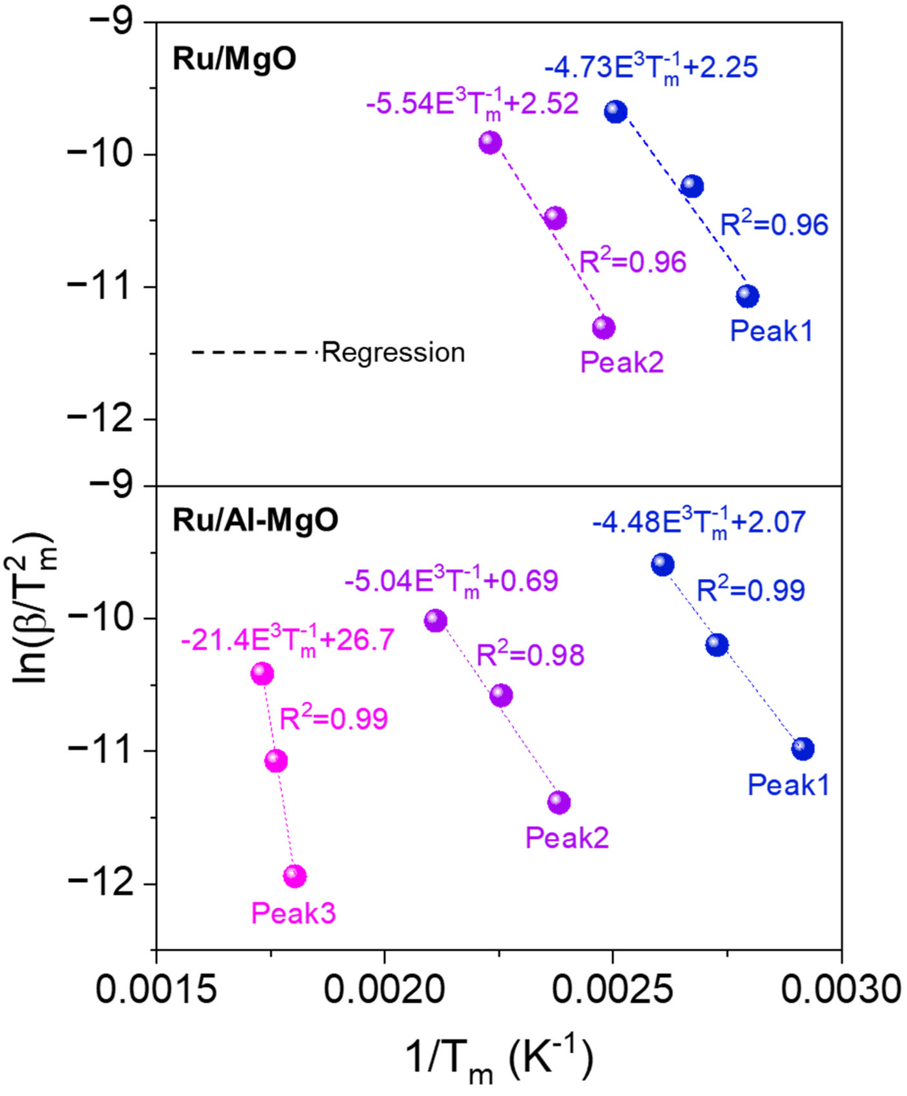

Supplementary Fig. 11. Calculation of the activation energies and the pre-exponential factors. Kissinger plots and calculated regressions for each HD production peak for Ru/MgO and Ru/Al-MgO specimens.

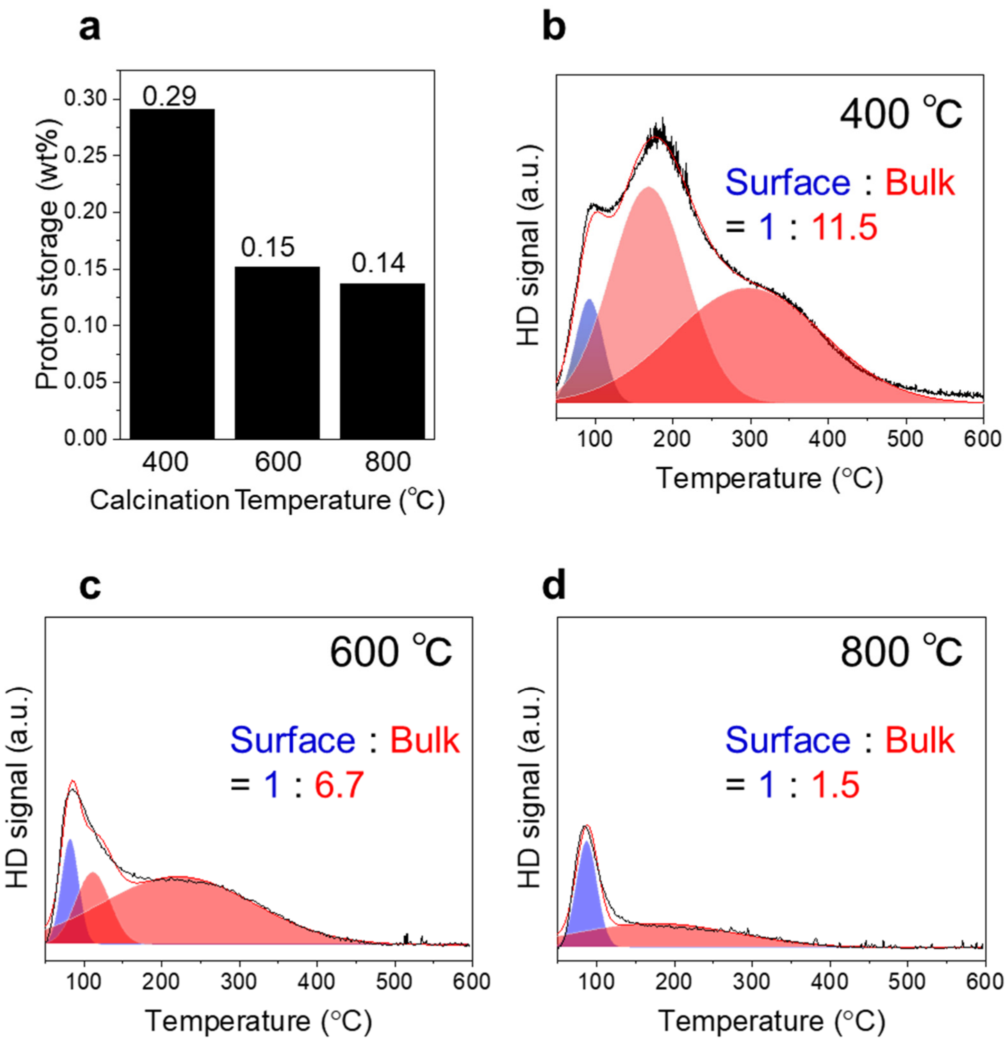

Supplementary Fig. 12. H^+ diffusion property of Al-MgO calcined at different temperature.

a H^+ storage capacities of Al-MgO calcined at different temperature based on TG measurements. **b, c, d** HD production during H_2-TPD following D_2 annealing from Ru supported Al-MgO calcined at different temperature as obtained by mass spectrometry.

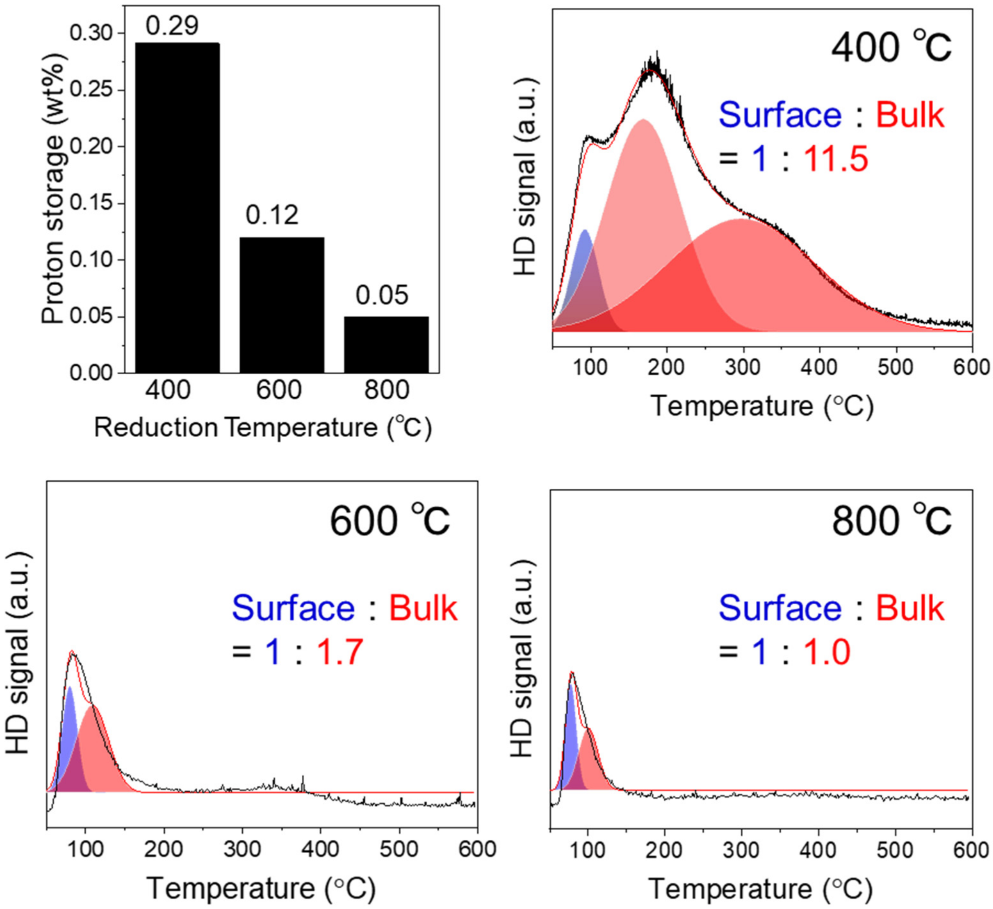

Supplementary Fig. 13. H^+ diffusion property of Al-MgO reduced at different temperature.

a H^+ storage capacities of Ru/Al-MgO reduced at different temperature based on TG measurements. **b, c, d** HD production during H_2-TPD following D_2 annealing from Ru supported Al-MgO reduced at different temperature as obtained by mass spectrometry.

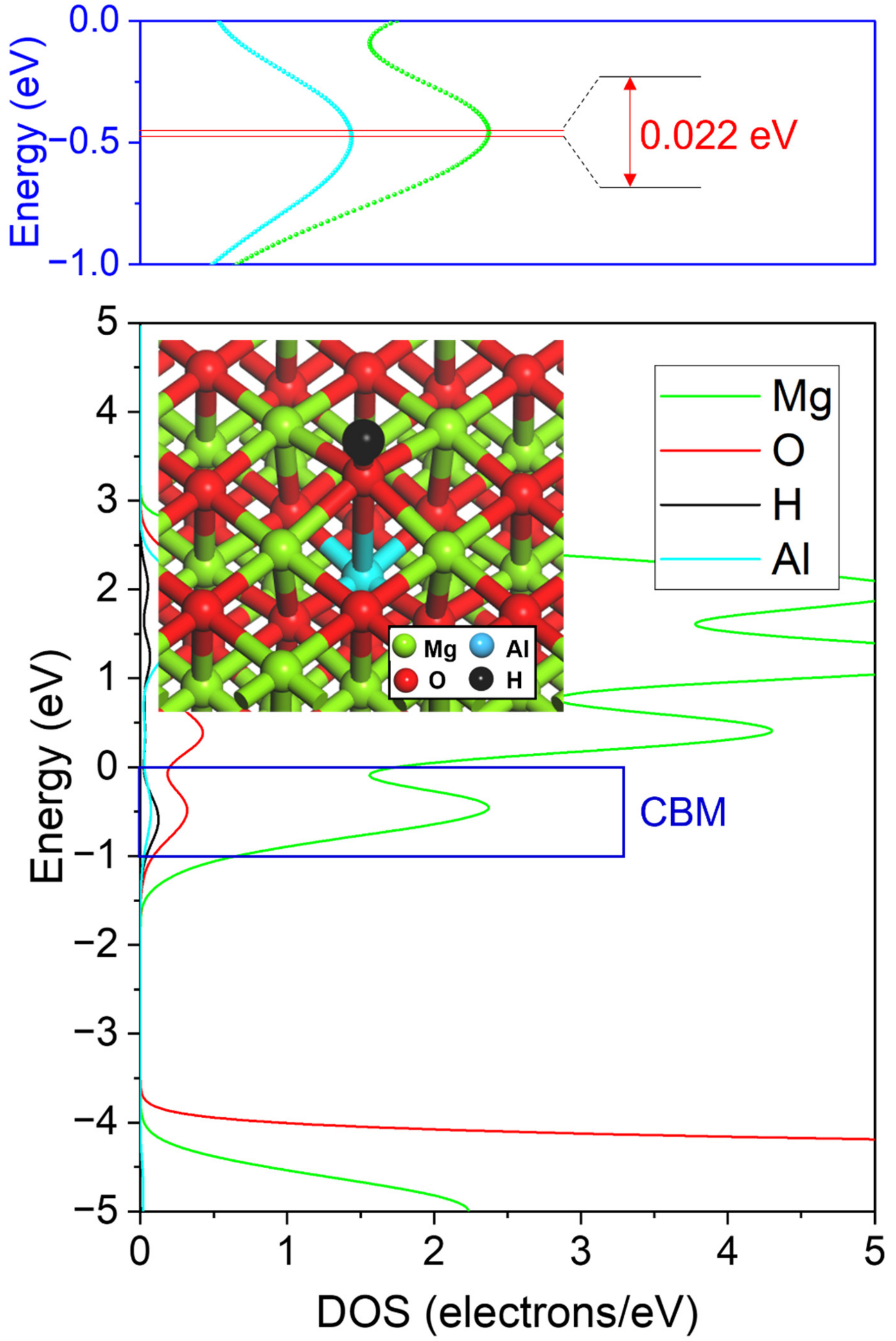

Supplementary Fig. 14. The effect of Al position on the PDOS.

Partial density of states results obtained using inset model, which contains single Al_{Oh} within bulk MgO. An enlarged view of the conduction band minimum is provided in the upper part of the figure.

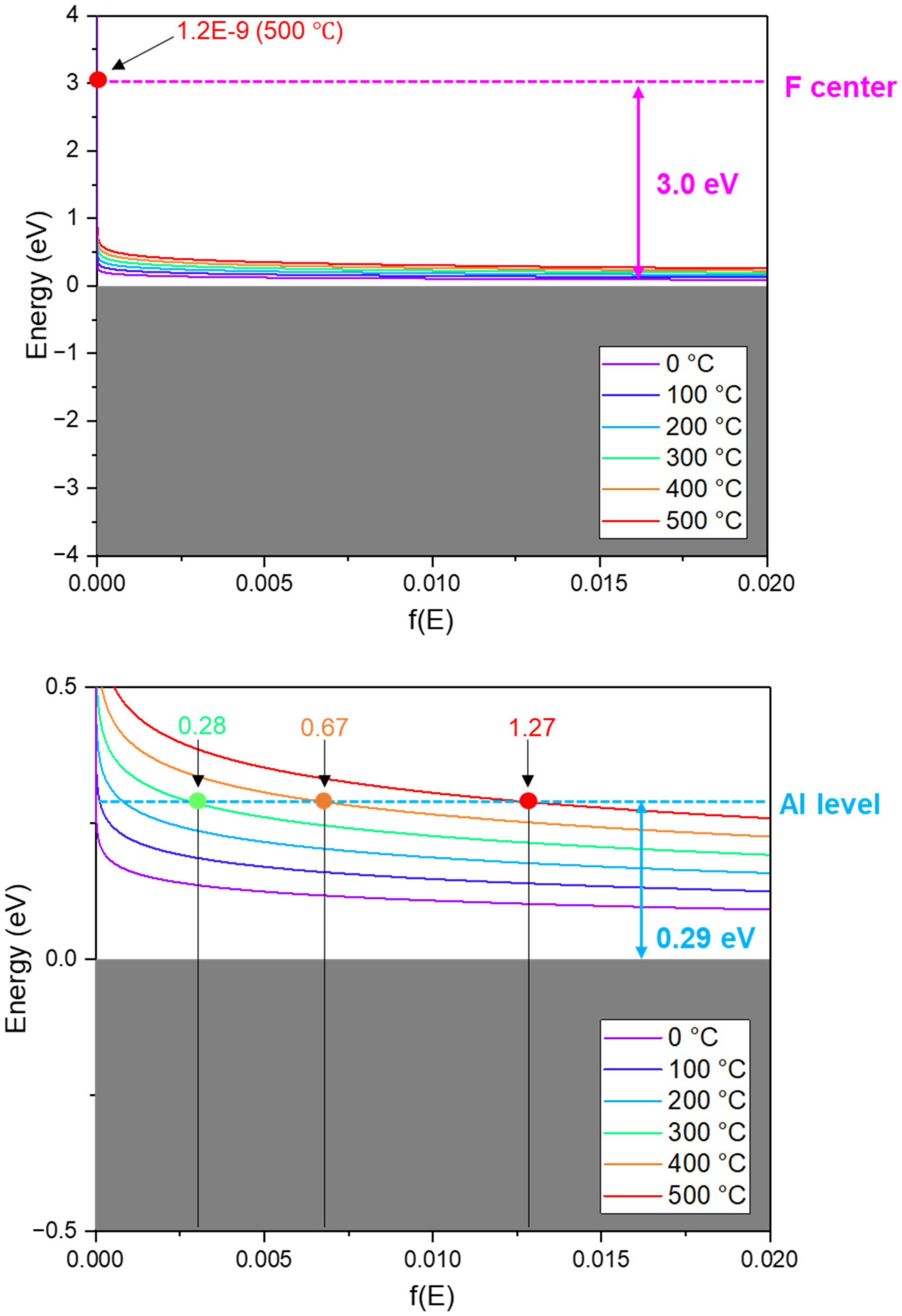

Supplementary Fig. 15. Fermi distribution functions calculated for different temperatures. The top and bottom figures show results for energy regions ranging from -4 to 4 eV and from -0.5 to 0.5 eV together with various band gaps between the conduction band minimum (CBM) and an estimated F center (3.0 eV) or Al level (0.29 eV). Excitation probabilities from the F center (top) and Al level (bottom) to the CBM at each temperature are provided next to each circle symbol.

a

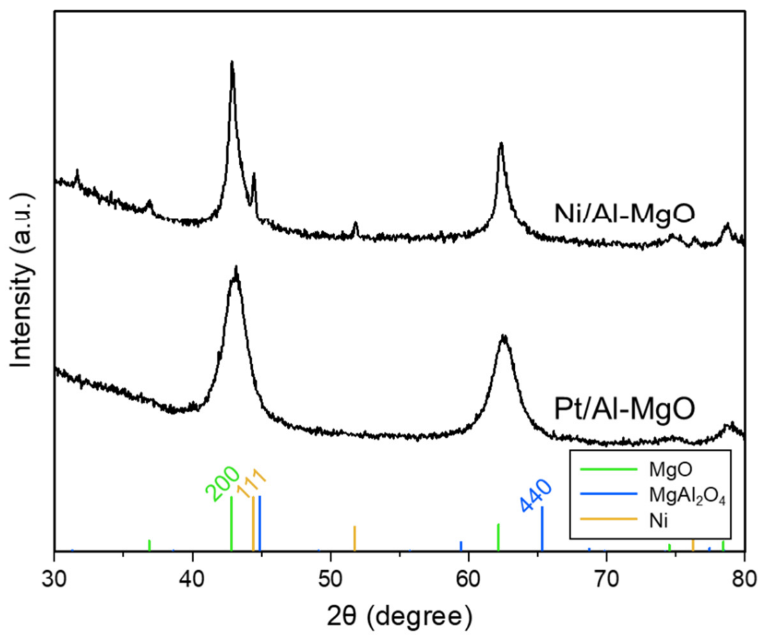

b

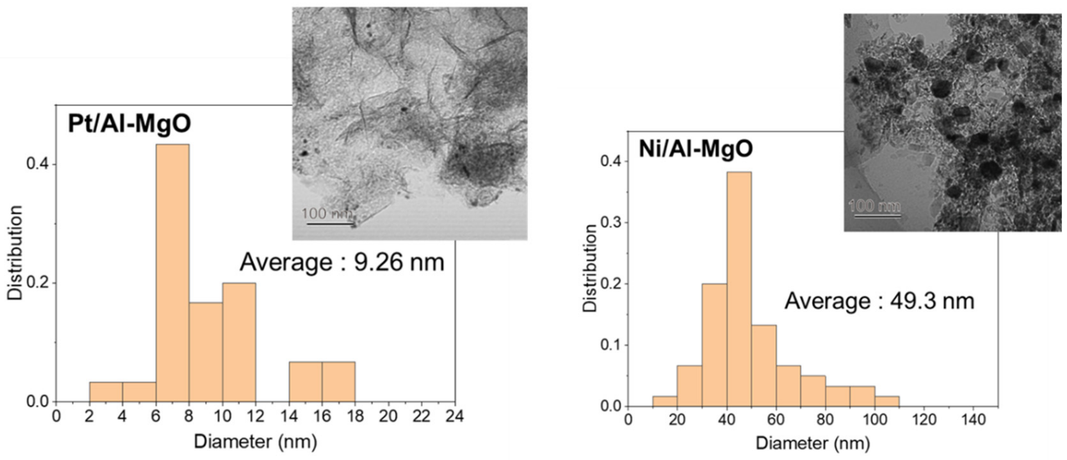

Supplementary Fig. 16. The structural characterizations of Pt/Al-MgO and Ni/Al-MgO.
a XRD patterns obtained from Pt/Al-MgO and Ni/Al-MgO. **b** TEM images obtained from Pt/Al-MgO and Ni/Al-MgO.

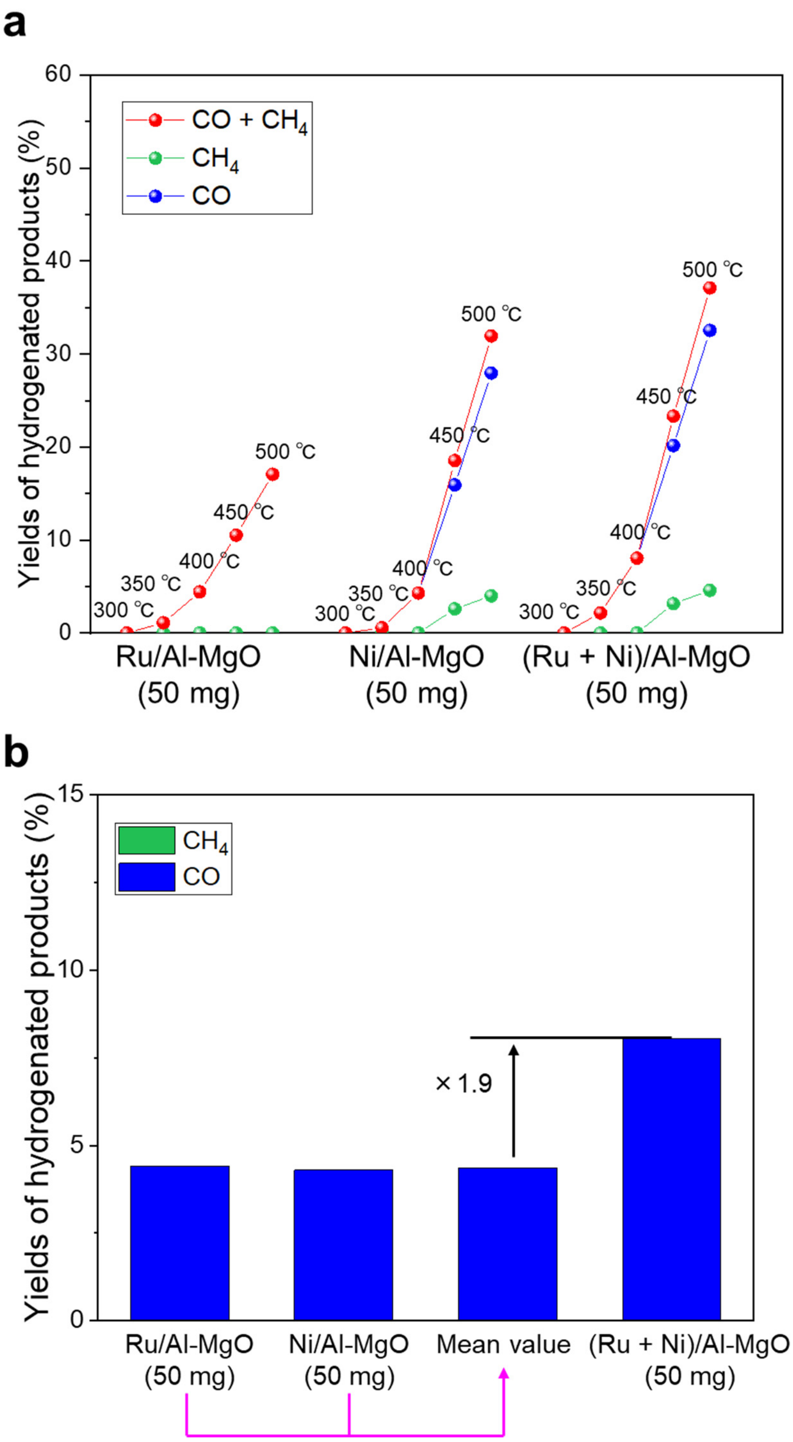

Supplementary Fig. 17. The performance of catalysts employing Ru as a H_2 dissociation site. **a** Yields of hydrogenated products obtained from Al-MgO catalysts loaded with Ru, Ni or (Ru + Ni) at various temperatures. **b** Catalytic CO_2 conversion to CO and CH_4 at 400 °C for (i) 50 mg of Ru/MgO, (ii) 50 mg of Ni/MgO, (iii) average of values for Ru/MgO and Ni/MgO, and (iv) 50 mg of (Ru + Ni)/MgO.

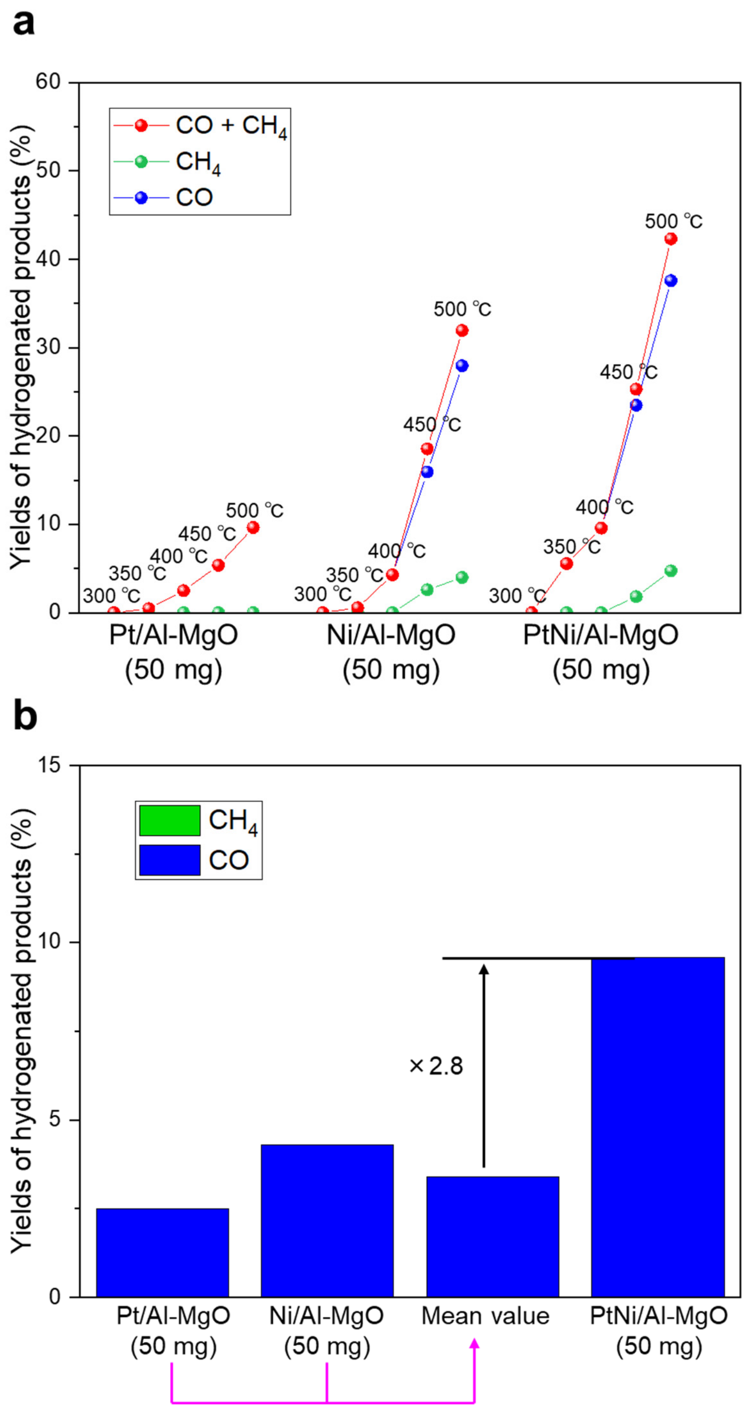

Supplementary Fig. 18. The performance of a Pt and Ni co-supported catalyst.

a Yields of hydrogenated products obtained from Al-MgO catalysts loaded with Pt, Ni or PtNi at various temperatures. **b** Catalytic CO_2 conversion to CO and CH_4 at 400 °C for (i) 50 mg of Pt/Al-MgO, (ii) 50 mg of Ni/Al-MgO, (iii) average of values for Pt/Al-MgO and Ni/Al-MgO, and (iv) 50 mg of PtNi/Al-MgO.

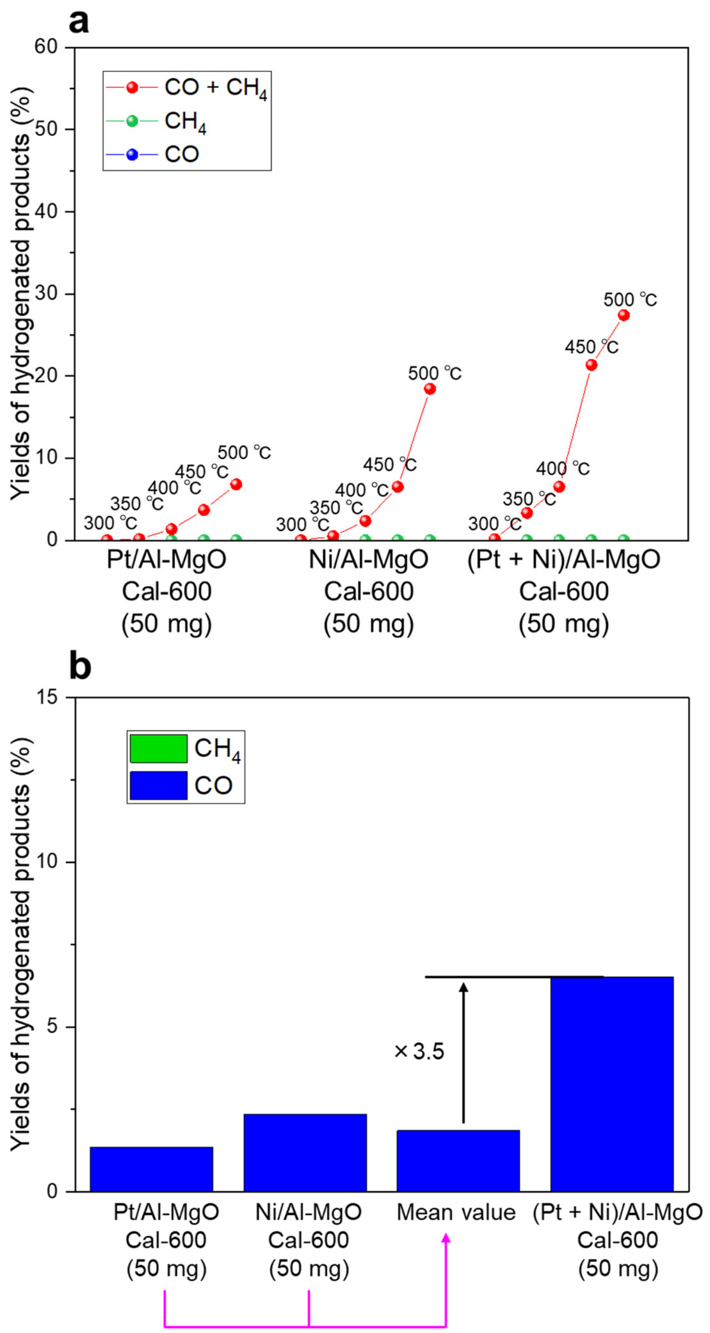

Supplementary Fig. 19. The performance of catalysts employing Al-MgO calcined at 600 °C. **a** Yields of hydrogenated products at various temperatures obtained from Pt, Ni or (Pt + Ni) catalysts employed Al-MgO calcined at 600 °C. **b** Catalytic CO_2 conversion to CO and CH_4 at 400 °C for (i) 50 mg of Pt/Al-MgO Cal-600, (ii) 50 mg of Ni/Al-MgO Cal-600, (iii) average of values for Pt/Al-MgO Cal-600 and Ni/Al-MgO Cal-600, and (iv) 50 mg of (Pt + Ni)/Al-MgO Cal-600.

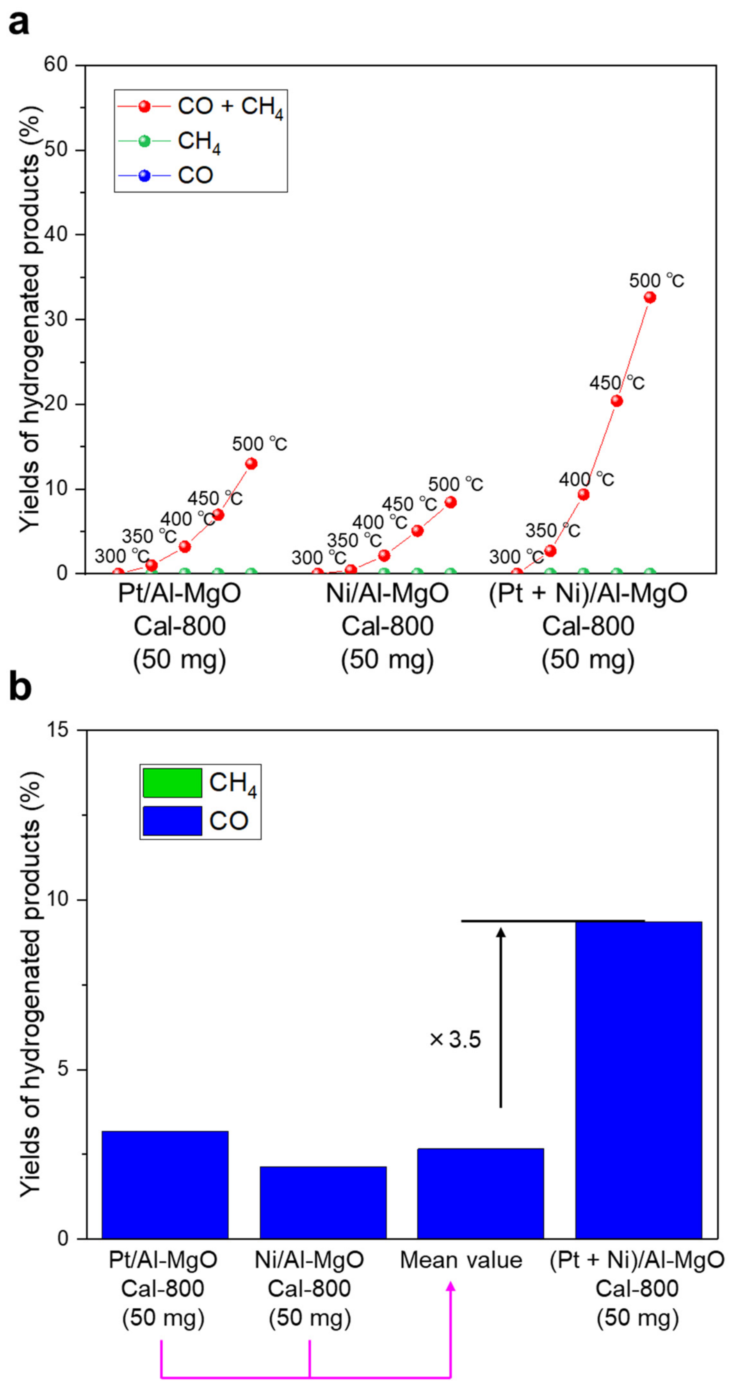

Supplementary Fig. 20. The performance of catalysts employing Al-MgO calcined at 800 °C. **a** Yields of hydrogenated products at various temperatures obtained from Pt, Ni or (Pt + Ni) catalysts employed Al-MgO calcined at 800 °C. **b** Catalytic CO_2 conversion to CO and CH_4 at 400 °C for (i) 50 mg of Pt/Al-MgO Cal-800, (ii) 50 mg of Ni/Al-MgO Cal-800, (iii) average of values for Pt/Al-MgO Cal-800 and Ni/Al-MgO Cal-800, and (iv) 50 mg of (Pt + Ni)/Al-MgO Cal-800.

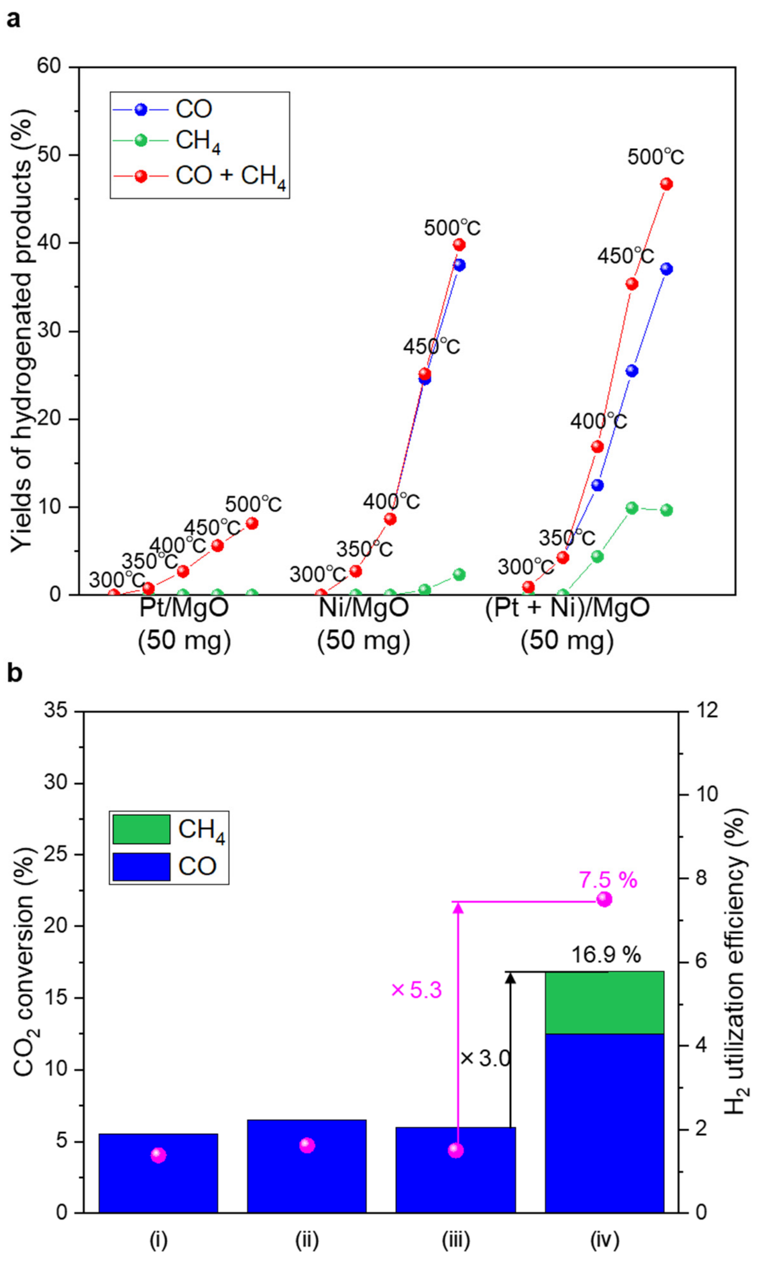

Supplementary Fig. 21. The performance of catalysts employing MgO.

a Yields of hydrogenated products obtained from MgO catalysts loaded with Pt, Ni or (Pt + Ni) at various temperatures. **b** Bars: catalytic CO_2 conversion to CO and CH_4 at 400 °C for (i) 50 mg of Pt/MgO, (ii) 50 mg of Ni/MgO, (iii) average of values for Pt/MgO and Ni/MgO, and (iv) 50 mg of (Pt + Ni)/MgO. Plot: H_2 utilization efficiencies during the CO_2 hydrogenation reaction.

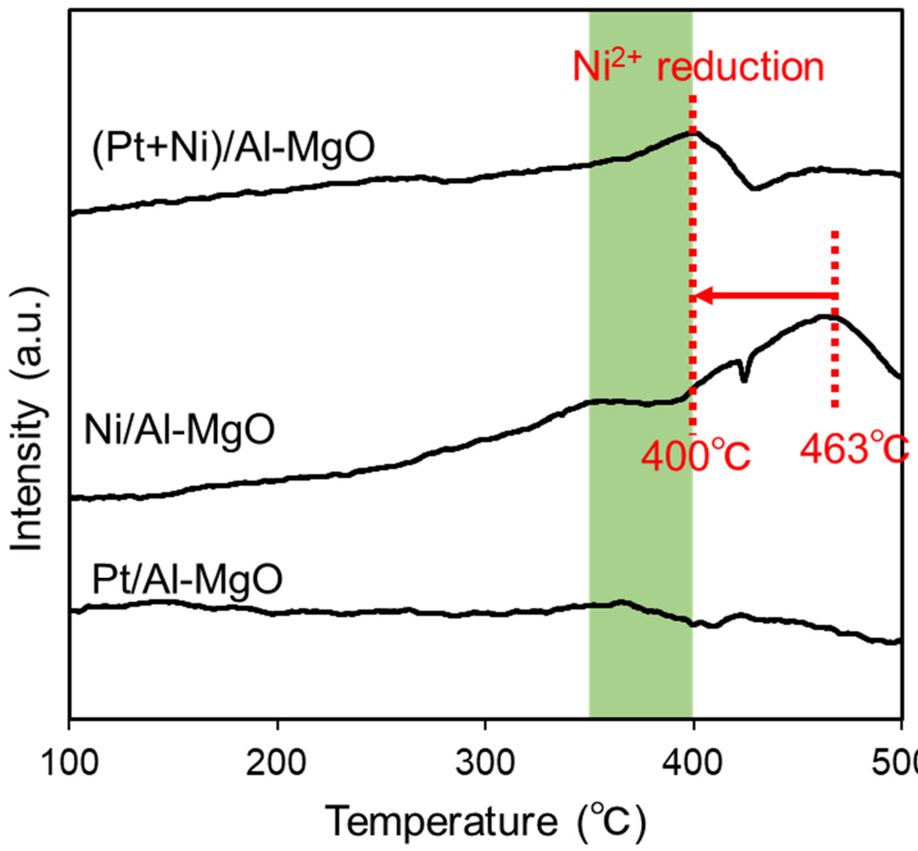

Supplementary Fig. 22. The enhancement of Ni^{2+} reduction by hydrogen spillover.

Hydrogen temperature programmed reduction profiles obtained for Al-MgO specimens loaded with Pt, Ni or (Pt + Ni). The temperature range over which the catalytic activity of the (Pt + Ni)/Al-MgO was significantly increased is indicated in green.

Supplementary References

1. Van Orman, J. A., Li, C. & Crispin, K. L. Aluminum diffusion and Al-vacancy association in periclase. *Phys. Earth Planet. Inter.* **172,** 34-42 (2009).
2. Rebours, B., d'Espinose de la Caillerie, J.-B. & Clause, O. Decoration of Nickel and Magnesium Oxide Crystallites with Spinel-Type Phases. *J. Am. Chem. Soc.* **116,** 1707-1717 (1994).
3. Seto, Y. & Ohtsuka, M. ReciPro: free and open-source multipurpose crystallographic software integrating a crystal model database and viewer, diffraction and microscopy simulators, and diffraction data analysis tools. *J. Appl. Crystallogr.* **55,** 397-410 (2022).
4. Ichikawa, S., Miyazawa, K. i., Ichinose, H. & Ito, K. The microstructure of deformed nanocrystalline Ag and Ag/Fe alloy. *Nanostruct. Mater.* **11,** 1301-1311 (1999).
5. Wang, S. et al. Activation and Spillover of Hydrogen on Sub-1 nm Palladium Nanoclusters Confined within Sodalite Zeolite for the Semi-Hydrogenation of Alkynes. *Angew. Chem. Int. Ed.* **58,** 7668-7672 (2019).
